# Factors impeding the acceptability and use of malaria preventive measures: implications for malaria elimination in eastern Rwanda

**DOI:** 10.1186/s12936-015-0659-6

**Published:** 2015-03-31

**Authors:** Chantal Marie Ingabire, Alexis Rulisa, Luuk Van Kempen, Claude Muvunyi, Constantianus JM Koenraadt, Michele Van Vugt, Leon Mutesa, Bart Van Den Borne, Jane Alaii

**Affiliations:** Department of Health Education & Promotion, Maastricht University, Maastricht, The Netherlands; Medical Research Center, Rwanda Biomedical Center, Kigali, Rwanda; Radboud University Nijmegen, Nijmegen, The Netherlands; College of Medicine and Health Sciences, University of Rwanda, Rwanda, The Netherlands; Wageningen University, Wageningen, The Netherlands; Academic Medical Center, Amsterdam, The Netherlands; Context Factor Solutions, Nairobi, Kenya

**Keywords:** Community engagement, Malaria elimination, Bedbugs, LLIN, IRS, CBHI, Health care seeking, Rwanda

## Abstract

**Background:**

Long-lasting insecticidal nets (LLIN), indoor residual spraying (IRS) and malaria case treatment with artemisinin-based combination therapy (ACT) have been proven to significantly reduce malaria, but may not necessarily lead to malaria elimination. This study explored factors hindering the acceptability and use of available malaria preventive measures to better inform area specific strategies that can lead to malaria elimination.

**Methods:**

Nine focus group discussions (FGD) covering a cross-section of 81 lay community members and local leaders were conducted in Ruhuha, Southern Eastern Rwanda in December 2013 to determine: community perceptions on malaria disease, acceptability of LLIN and IRS, health care-seeking behaviours and other malaria elimination strategies deployed at household and environmental levels. Discussions were recorded in Kinyarwanda, transcribed into English and coded using Nvivo 10 software.

**Results:**

Participants ranked malaria as the top among five common diseases in the Ruhuha sector. Participants expressed comprehensive knowledge and understanding of malaria transmission and symptoms. The concept of malaria elimination was acknowledged, but challenges were reported. Sleeping under a bed net was negatively affected by increase of bedbugs (and the associated irritability) as well as discomfortable warmness particularly during the dry season. These two factors were reported as common hindrances of the use of LLIN. Also, widespread use of LLIN in constructing chicken pens or as fences around vegetable gardens was reported.

Participants also reported that IRS appeared to lead to an increase in number of mosquitoes and other household bugs rather than kill them. Prompt health centre utilization among participants with presumed malaria was reported to be common particularly among subscribers to the subsidized community-based health insurance (CBHI) scheme. In contrast, the lack of CBHI and/or perceptions that health centre visits were time consuming were common reasons for the use of over-the-counter medicines for malaria management.

**Conclusion:**

In this study, identification of behavioural determinants in relation to LLIN use, IRS acceptability and health care seeking is a critical step in the development of effective, targeted interventions aiming to further reduce malaria transmission and elimination in the area.

## Background

Malaria is one of the leading causes of mortality in Africa with about 90% of the 528,000 malaria deaths estimated globally, occurring in the African region [1]. As in many other sub-Sharan African countries, use of long-lasting insecticidal nets (LLIN), indoor residual spraying (IRS) in addition to malaria case treatment with artemisinin-based combination therapy (ACT) have been the major malaria control tools used in Rwanda with, a greater than 50% decline in malaria cases and deaths observed among children and adults [2,3]. In 2004, national malaria control programme (NMCP) adopted the home -based management of fever and treatment using ACT through trained community health workers (CHWs) and over 95% of all fever cases are tested and treated appropriately [4]. In addition, the country deployed IRS in highly malaria transmission areas since 2007 and has achieved a universal LLIN coverage (one net per two people) early 2011 [4,5].

To achieve a successful malaria pre-elimination programme, ensuring optimal healthcare for the population through access to highly sensitive diagnostic tests and effective treatment is essential [6,7]. In this framework, Rwanda established a community-based health insurance (CBHI) initiative since 2004 to provide subsidized health care to the majority of the population in informal sector. CBHI has led to increased health services utilization with more than 90% subscription noted by 2010 [8].

However, some areas in Rwanda including Ruhuha Sector (Bugesera District, Eastern Province) remain zones with high levels of endemic malaria despite the availability of malaria control strategies [9,10]. To assess the epidemiology of both symptomatic and asymptomatic malaria infections, two studies combining passive and active case detection methods were conducted in the area. Findings showed a 22% and 5% malaria prevalence at the health centre and household level respectively [9,10]. Availability of public health interventions is not enough to ensure optimum use [6]. In Rulisa and colleagues, 82% of those with LLIN had the LLIN hung up [9]. Hence, community acceptance of available interventions for malaria remains one of the key parameters if elimination is to be achieved [6]. To complement approaches deployed by the NMCP, including IRS, LLIN and ACT, a malaria elimination project for Ruhuha (MEPR) was initiated in 2012. MEPR has deployed a bottom-up approach to accelerate progress towards malaria elimination. The first component of this community-engagement strategy was deployment of an ‘open space’ approach and explored various ways in which the local community, as primary stakeholders, could contribute to malaria elimination [11]. To generate further insight, a quantitative baseline survey among 4,705 households was conducted in combination with a qualitative study using focus group discussion (FGD) methodology. This paper reports findings from the qualitative study that aimed to explore a detailed contextual perspective on the acceptability and use of malaria preventive measures to better inform area specific strategies that can lead to malaria elimination.

## Methods

### Study area

Ruhuha sector is part of Bugesera district, eastern province of Rwanda. The area covers 54 km^2^ and is surrounded by five marshlands in which agriculture (mainly rice farming) is commonly practiced. The total population is estimated at 21, 606 individuals.

### Selection and enrolment of participants

Maximum variation sampling was used to identify shared experiences across individuals representing a wide variation of interest, following procedures described elsewhere [12]. Nine FGDs were conducted in December 2013, with a total of 81 participants involved. A cross section of participants was purposively sampled based on their role in the community following a stakeholder analysis exercise previously conducted. To get entry to the target population, an invitation letter was sent through local authorities. Groups included (1) rice farmers cooperatives, (2) agricultural cooperatives (bee-keepers, livestock farmers, pineapples cultivators), (3) various other cooperatives such as transportation, sewing, security and traders cooperatives among others, (4) community health workers (CHWs), (5) health care professionals, (6) general community members, (7) school teachers, (8) youth and (9) local and religious leaders.

### Discussions domains and procedures

Discussion domains across the FGDs included perceptions on malaria related themes by community members including; acceptability of LLIN and IRS, health care-seeking behaviours and suggestions on malaria elimination strategies at both household and environmental levels.

### Conduct of interviews

Two PhD researchers, trained in moderation and notes taking skills, led discussions. The purpose of the discussions was explained and verbal informed consent obtained from participants. Discussions were all conducted in the local language, Kinyarwanda, and lasted about two hours each.

### Ethical approval

The National Health Research Committee and the Rwanda National Ethics Committee approved the study protocol (NHRC/2012/PROT/0015 and No. 385/RNEC/2012).

### Data processing

Discussions were taped and recorded in Kinyarwanda. A team of two PhD researchers transcribed verbatim and translated the recordings into English for further analysis using Nvivo software, version 10 (produced by QSL international). The same team created a framework of codes for emerging themes after reviewing the transcripts, conducted independent open coding sessions for similar data sections and compared their findings for reliability purposes.

## Results

### Demographic description

Altogether 81 participants were involved in this study. More than half (54%) of the participants were men with participants’ ages ranging from 25 to 53 years but with the majority (80%) aged less than 40 years as shown in Table 1.

**Table 1:** **Source population Background characteristics**

**Characteristics**		**Respondents [N, (%)]**
**Sex**	Male	44 (54.3)
**Age**	Female	37 (45.6)
	Between 25 and 53 year-old	44 (54.3)
	Older than 53 year-old	37 (45.6)
Education level*	Primary	37 (45.6)
	Secondary	34 (41.9)
	University	10 (12.3)
**Group session**	Local and religious leaders	9 (11.1)
	Health care professionals	7 (8.6)
	Youth	9 (11.1)
	School teachers	8 (9.8)
	Community members	10 (12.3)
	CHWs	10 (12.3)
	Rice farmers cooperatives	10 (12.3)
	Mixed agricultural cooperatives	10 (12.3)
	Transport, security and sewing cooperatives	8 (9.8)

*Partial or completed.

### Local perceptions of malaria

Malaria, tuberculosis, human immunodeficiency virus (HIV), pneumonia, diarrhoea, and malnutrition were all reported to be the most common observed disease conditions in the community but with malaria cited as the most commonly observed. The local name for malaria is “*malariya”*. Clinical malaria severity was described according to the preserved degree of severity called as “*malariya y’igikatu*” or *malariya y’igisore*” with the two terms used interchangeably when referring to signs such as high fever, shivering and convulsions. A few participants identified a peculiar malaria clinical presentation locally termed “*malariya y’umuhindo*” referring to the difference in time periods when people suffering tend to feel well (during the day), but have severe symptoms (at night).

Participants perceived malaria as a serious threat to their lives and the quality of their life. As frequently mentioned, malaria affects the whole family including parents and children with repeated malaria episodes among school children reported to lead to low school attendance, thus negatively impacting school performance. In addition, reduced productive days for parents who were sick lead to a high family expenditure and may negatively impact household social economic status. Malaria attacks in a parent was associated with major consequences for the family; children became caregivers and missed classes as result.

It was also reported that during malarial episodes, expenditures for diagnosis and treatment tended to increase particularly for the family without the subsidized CBHI with this associated with delays in seeking health care.

*Malaria affects the economy of families in terms of money spent for treatment and caring for a sick person; and the burden of expenses may not be on the family alone, but even the sector and the nation at large, because the budget spent on malaria treatment would have been spent on other developmental projects like roads or schools etc.* Male, 47 yrs. FGD religious and local leaders.

*When a man or woman in the family gets infected with malaria, he or she will not work and so the family’s economy is affected…even when children fall sick, the parent will stop other activities so as to take the child for treatment and the time lost may affect the family’s economy.* Female, 28 yrs. FGD youth.

### Knowledge of the cause of malaria and symptoms

Malaria was perceived to be transmitted by a mosquito by the majority although not all study participants were aware of the type of mosquito that transmits the *Plasmodium* parasite; only a few number reported knowing a female mosquito, termed “*Anopheles*”. Participants cited seasonal weather variations with rainy seasons associated with high mosquito proliferation.

*Mosquitoes that transmit malaria grow and multiply much in this region mainly due to hot weather that is conducive to their proliferation.* Male, 40 yrs. FGD religious and local leaders.

*Bugesera is a region surrounded by many water bodies and forest; in addition it is hot in most of the seasons with temperature of about 31*°*C recorded during the dry season. These factors are conducive for breeding and multiplication of mosquitoes; hence the reason for malaria becoming endemic in Bugesera*. Male, 54 yrs. FGD religious and local leaders.

Other participants reported human behaviours such as household practices including the keeping of water tanks uncovered and failing to clear stagnant water bodies as determinants of malaria transmission.

*It’s true, I think that is why in the previous months of October and November malaria cases are many because we are surrounded by many food crops that may act as breeding sites for mosquitoes. There are also pits made by brick makers near our villages that collect water during rain, which become good breeding sites for mosquitoes*. Male, 33 yrs. FGD transportation cooperatives.

In addition to knowledge on how malaria is transmitted in the community, participants had wide knowledge on malarial symptoms with fever, nausea, headache and shivering reported as the most observed symptoms. Convulsions were associated with presumed severe malaria.

### Malaria preventive measures adopted by the local community

Malaria was perceived as not inherited rather acquired in all accounts and thus preventable. Preventive measures cited as already in use included LLIN and IRS, closing windows and doors early in the evening, clearing mosquito breeding sites and seeking early healthcare after first symptoms emerged.

*Malaria is preventable. The strategies for preventing malaria include keeping good sanitation around our homes by cleaning the surroundings, clearing bushes, closing windows early in evening, avoiding staying for long outdoor in late evening and sleeping under mosquito net always at night*. Female, 41 yrs. FGD rice farmers cooperatives.

### Mosquito nets

Although emerging themes on LLIN use included acceptability, factors influencing their use were also described by participants, such as knowledge, seasonality, adverse events, perceived insufficient number of LLIN distributed and bedbug infestation.

### Acceptability of LLIN

All participants agreed on the effectiveness of LLIN when used correctly every night. There were no clear differences among people who commonly used LLIN on a household level. Some participants had a preference for the type of nets distributed with the rectangular type of LLIN less preferred to the more common distributed circular type. Rectangular LLIN are hung at all four corners of a bed while rounded ones are simply hung in the middle of the bed then lowered and tucked under the mattress at bedtime. When there is no bed, a mattress is laid on a mat by the floor and the net hung over the mattress whist being suspected by a long string. LLIN were perceived to prevent malaria and reduce mosquito-biting frequency, provide warmth during rainy seasons, and prevent snakebites.

*When I am sleeping under a bed net, I feel like I am in paradise; because I feel relaxed, no fear of mosquito bites, no need of covering myself with a blanket; I just lie as a king in a palace*. Female, 33 yrs. FGD transportation cooperatives.

*Also children are protected from cold when they sleep under bed nets, as they are mostly uncovered at night*. Male, 40 yrs. FGD Rice farmers cooperatives.

### Factors impeding effective use of LLINs

#### Knowledge

A few participants stated that in some households, LLIN are not consistently used despite owning them because some community members might not perceive malaria as a burden and/or preferred to use LLIN for other activities needed for daily survival including building of chicken pens and home kitchen gardens and fishing.

#### Seasonality

Participants underscored the role of weather as one of the reasons for inconsistent LLIN use. Use of LLIN in the dry season was associated with increases in the level of warmth and uncomfortable for some of the participants. LLIN were therefore more frequently used during rainy seasons when the temperature is relatively low.

*Some people dislike bed nets because they increase the temperature and lead to sweating at night hence prefer sleeping without them*. Female, 28 yrs. FGD general community members.

#### Adverse events

A few cases of itchy skin rashes were reported to occur among especially those using new LLIN. Some participants reported that instructions were needed on how new LLIN should be handled before hanging them for the first time. Secondly, some community members believed that once an LLIN is washed it is no longer effective.

*Some people develop rashes when they use bed net soon after opening it from its package especially when used before washing*. Female, 41 yrs. FGD general community members.

*I also had a reaction when I used it immediately after getting it from the health centre. I developed severe rashes and swelling of the face and on reporting this at the health centre, I was told to wash the net with water and dry it for about two hours before using it; I did exactly what I was told and the problem never occurred again*. Male, 53 yrs. FGD general community members.

#### Coverage of LLIN

LLIN were reportedly received by CHWs during mass campaigns and distributed at household even though some of the participants reported that generally, the number of LLIN distributed at the household level does not always correspond to the number of beds or sleeping places in the house and hence not all household members are able to use LLIN. Participants argued that it is common for some community members who appreciated the value of LLIN to buy nets from poorer families who opt to sell their nets at a very low price in order to get funds to pay for other basic needs.

*Sometimes when CHWs come to supply bed nets too few are provided hence some families will have some beds remaining uncovered*. Female, 49 yrs. FGD general community members.

#### Bedbug infestation

Across all the interviews, the presence of bedbugs was frequently mentioned as a major hindrance of the use of LLIN over the last two years and this may be an important factor in the recent increase in malaria. Bedbugs tend to hide in cracks and crevices of a bed, house walls as well as the top inside of the net where they lower down to bite during the night. Bedbugs were reportedly to affect almost all houses in this community and were reported to easily spread between homes. Bedbugs are reported to have a very aggressive bite that leads to pruritic rashes. Although participants reported initially that bedbugs infestation was perceived to be associated with poor sanitation, it was later noted that this was not the case. Bedbugs originated from places with dense populations of people such as boarding schools, prisons, solidarity camps and hotels. Some community members also perceived bedbugs to be a result of IRS targeting mosquitoes in the area, claiming that IRS tends to cause an increase in frequency of bedbugs.

O*ne reason why people don’t use bed nets nowadays is due to the problem of bedbugs; these insects climb into the nets and bite people sleeping in the nets and as a result many people have decided to remove bed nets*. Male, 40 yrs. FGD general community members.

*About 90% of homes have bedbugs; these insects multiply rapidly if you pick one from a neighbor, within a short time they will have occupied the whole house*. Male, 30 yrs. FGD transportation cooperatives.

*I think it is not possible to eliminate bedbugs completely; because if I managed to chase them away today as many have said, tomorrow the neighbour who did not attempt to fight them will bring them back to the house and besides I cannot avoid visits from neighbours. Unless all homes fight them at the same time, which is not an easy thing since people have different programmes in their families, it will not be easy to control them.* Male, 33 yrs. FGD transportation cooperatives.

Strategies that were taken by some homes, but with limited success in control of bedbugs included splashing hot water mixed with a locally available detergent powder, OMO® onto interior walls of homes. This was deemed successful if combined with soaking all bed linen and clothes in the same combination of hot water and OMO®. However, this technique was found to be most effective on walls made of cement mainly with bedbugs noted to hide in small crevices existing in the mud-made-walls that rendered the technique not effective for use on mud walls. Another strategy for fighting bedbugs was the use of chemicals bought in shops selling agricultural fertilizers. The chemicals locally termed “s*imikombe*” (diazinon) mixed with hot water and splashed to the walls were also reported to kill off bedbugs.

These bedbug-control methods were however not widely known and/or use by many participants implying that a large part of the community was not aware of how to fight bedbugs. Participants highlighted a need of coordinated efforts including community sensitization to apply specific strategies simultaneously to prevent resurgence.

*I tried to use both the medicine and boiled water mixed with OMO, removed everything inside the house and washed them and since March, I have not seen any more bedbugs in my house*. Female, 35 yrs. FGD school teachers.

*Generally, there is no effective method available for fighting bedbugs that suites all. Even community members who managed to control them [bedbugs] using hot water and OMO could be those who have houses with smooth walls and cemented floor; but remember the majority of our people live in mud houses with rough walls and un-cemented floors where bedbugs can hide and multiply. And as you may know, these insects are stubborn and difficult to kill and even if only a few eggs remains they will hatch into larvae that soon mature into adults. I think it requires a more effective chemical method that can be sprayed inside houses to kill all bedbugs without leaving even eggs*. Male, 54 yrs. FGD religious and local leaders.

*It requires regular efforts, otherwise the resurgence is high. We may do it every two weeks or every month. It is also better to mention this problem whenever there is a meeting with community members. The same way, local authorities discuss about health problems, they also need to pay attention to the problem of bedbugs*. Female, 27 yrs. FGD school teachers.

### Indoor residual spraying

All participants were aware of the IRS programme with and almost all houses in the area being sprayed about twice a year. However, the feelings about IRS were ambiguous and sometimes contradictory. Some participants highlighted the importance of IRS in the fight against mosquitoes while some reported negative perceptions about IRS use. A reported strong repugnant smell associated with IRS was reported as a deterrent of IRS use. Some participants reported developing allergic reactions including asthma and swelling of the face on entering a sprayed house. A few participants had concerns that the product used may no longer kill mosquitoes but rather seemed to activated fleas, mites and mostly bedbugs.

Participants mentioned that the perceived ineffectiveness of the IRS was also attributable to the fact that not all rooms inside a house are always sprayed with some participants reporting that the bedroom and storage rooms were usually unsprayed due to cultural norms and insecurity with strangers not allowed to enter the sleeping room or get to know the content of the storage rooms respectively. A small number of participants mentioned that the people selected to perform the spraying are sometimes not known in the local area and suggest that this may be the reason for the mistrust.

*Generally there is no problem I ever encountered associated with the spraying of this chemical. Even those who say that it is ineffective, it is because they do not allow the whole house to be sprayed mainly due to ignorance. IRS is very effective when people allow for spraying of all rooms and observe all regulations provided*. Male, 39 yrs. FGD rice farmers cooperatives.

*That chemical [(used in IRS] does not cause bed bugs and fleas, instead it activates them; they would have been there in the house dormant but after spraying the chemical to kill mosquitoes, they become activated.* Male, 33 yrs. FGD youth.

## Health care-seeking behaviours

### CBHI and health facility use

Many participants reported that the health centre was the first stop for care when someone is sick. The eases of visiting the health centre was attributed to CBHI membership that enabled them to seek health care as early as possible and at an affordable cost in view of the subsidized rates among those with CBHI membership.

*When you have a health insurance, you are not afraid of seeking health care hence a disease does not turn into a serious stage*. Female, 33 yrs. FGD youth.

*When I am sick and go to the health centre, I pay only 10% of my medical bill while the rest is paid by the community health insurance scheme. Even when they cannot treat you and a transfer to district hospital is required, you only pay a small portion of the whole treatment and ambulance services.* Male, 45 yrs. FGD rice farmers cooperatives.

### Use of over-the-counter medicines

Self-treatment including use of over-the-counter medications was the most common practice among those who did not possess CBHI and therefore were not able to afford an initial diagnosis. Some people cited barriers such as time constraints including delays at the health centre, due to long service queues, hence their preference for over-the-counter medications.

*Some buy* over-the-counter medicines *due to lack of “mutuelle”* [CBHI] *that would enable them to go to the health centre. Others say they do not have time of going to the health centre saying that they would spend the whole day waiting for treatment, hence they resort to purchasing over- the-counter medicines and this is common among people who are inherently busy such as business men.* Male, 33 yrs. FGD youth.

A few participants mentioned that frequent stock outs of medicines including antimalarial were reasons for non-attendance to health facilities with people tending to purchase over-the-counter medicines at a relatively high price (4,500 Rwfs (an equivalent of 7 USD) per dose).

### Resort to traditional medicine/healers

Participants suggested that people might occasionally seek the services of a traditional healer particularly when symptoms such as high fever coupled with convulsions are observed, as these symptoms were sometimes associated with being bewitched, and not necessarily linked to malaria.

*Sometimes malaria may come very severe and is then associated with convulsions and loss of conscience; some people may think that they have been bewitched and decide to go for witchcraft.* Male, 28 yrs. FGD transportation cooperatives.

A variety of herbal medicines were also reported as being used by some community members including “umugombe” (*Chenopodiaceae*), “umuravumba” (*Tragia brevipes pax*), “ubushohera”(*Basellaceae*), and “ibicucu” (*Manihot*) and umunyuragisaka” (*Esteraceae*).

### Effect of socio-economic categorization

The socio-economic categorization of Rwandans was started in 2011 and has had an impact on the amount that a family must contribute to obtain CBHI, which is related to the family’s wealth category. However, not everyone in the community is able to make timely contributions to the CBHI scheme for their entire family.

A FGD was held with local authorities and established that the Ministry of the local government was aware of the challenges that the socio-economic categorization was posing to subscription to CBHI and hence its impact on care seeking. They reported that a plan to revise the categorization in the near future was underway even as they reported that the premium CBHI costs were reasonable relative to the services offered to members nationwide.

*Mutuelle begun with a payment of 600 Rwf (0.86 USD) per family member, later it went up to 1,000 Rwf (1.44 USD) but now it stands at 3,000 Rwf (4.34 USD) per family member, which in fact is too high for many people to afford. For instance one person with six family members is required to pay 18,000 Rwf per year and few can afford it. We would suggest a reduction in the contribution to the Mutuelle to enable people afford malaria treatment.* Male, 38 yrs. FGD rice farmers cooperatives.

*Paying 3,000Rwfs (4.34 USD) is not much when looking at how the health units are paid well and on time, something that wouldn’t have been possible if the charges had not been raised. We only face difficulties in relation to the socio-economic categorization*. Male, 54 yrs. FGD religious and local leaders.

Despite the challenges faced by some community members when adhering to the CBHI scheme, community initiatives to facilitate access to the scheme were cited such as the full or partial contribution of the premium by cooperatives on behalf of their members.

### Malaria elimination

Participants described malaria elimination using various expressions including “*kurandura*” “uprooting” malaria in all homes by deploying all possible measures of malaria prevention. They suggested that malaria elimination was possible if all people were involved including lay members, local leaders, health care workers and religious leaders. All participants expressed their willingness to join the campaign, because malaria was perceived as a significant health burden in the area. Several malaria elimination strategies at multiple levels were suggested.

### Malaria elimination at a household level

Elimination of malaria at a household level required the application of available preventive vector control measures according to the participants. These included clearing of places where mosquitoes could hide and breed and always sleeping under LLIN. Participants felt they should be involved themselves as an example. In addition, the participants said that they had been recommended as parents to observe the proper use of LLIN by their children and ensure early treatment whenever a member of their family developed malaria symptoms. They stated that family members should also be encouraged to go for proper treatment and avoid buying over-the -counter medicines for self-treatment.

*All homes should remove places where mosquitoes can hide and breed, and sleep under bed nets always. In addition to that, people should be encouraged to close windows and doors early in the evening so that mosquitoes from the kraals do not fly into the houses to bite people at night.* Male, 35 yrs*.* FGD health professionals.

### Malaria elimination at a community level

Participants suggested that a multi-sectoral collaboration involving local leaders, farmers, religious leaders and others were critical in achieving malaria elimination. Community works, locally known as “*umuganda*” in which clearing bushes around homes and draining stagnant water in all villages was done monthly were reported as important.

From the youth and school FGD, there was an idea of forming anti-malarial clubs aiming at sensitizing area members on malaria prevention. Such clubs would be expected to be effective by organizing sports competitions such as football or other games to attract local people. In order to establish those clubs, however, materials such as radios, microphones, games equipment and charts as well as stationary would be needed for organizing messages to be conveyed to people in addition to a detailed training on malaria prevention strategies for the club members.

*To eliminate malaria at community level, all villages should work together through communal work and clear all bushes in their villages, drain stagnant water and conduct regularly meeting with all community members to sensitize them about sleeping under bed nets always.* Female, 34 yrs. FGD youth.

*There should be inter-sectoral collaboration in fighting malaria. The Ministry of Health should not be left to work alone in fighting malaria and even other sectors like local leaders, farmers, religious leaders should get involved vigorously together with all citizens collectively, otherwise, the Ministry of Health cannot succeed alone.* Male, 38 yrs. FGD health professionals.

*As youth, we could organize anti malaria clubs aimed at sensitizing people about malaria prevention.* Female, 25 yrs. FGD youth.

### Malaria elimination at a government level

Participants recommended that LLIN be availed to all households. A strong public and private partnership should be established to ensure that over-the-counter medicines are only provided to those who have been tested and confirmed to have malaria.

*I support the idea of allowing private drugstores in dispensing malaria drugs but I think it would be much better if they are first trained and provided instructions on the national malaria treatment protocol and should know which drugs of malaria to give and when*. Male, 34 yrs*.* FGD health professionals.

It was indicated that a large number of people using over-the-counter medicines are those without CBHI, implying that the government should create measures aimed at enabling the majority of people to pay for their CBHI.

Participants suggested that government should initiate measures for controlling mosquitoes by spraying marshlands, forests and dams, especially in a malaria endemic region like Bugesera district.

Finally, sensitization was reported to be an important tool that government could use to raise community awareness in malaria prevention strategies.

*Organizing shows on preventive measures on radio, television and making announcements is important especially when aired during evening hours when the messages reach a large number of the population.* Female, 39 yrs. FGD school teachers.

## Discussion

Malaria elimination requires optimal use of all available tools for prevention and treatment. Our study elicited mixed thoughts on the use of LLIN as one of these tools in the community. The benefits of LLIN use were recognized mainly as malaria prevention. However, a perceived decrease in the use of LLIN was highlighted related to their alternative uses, a recent emergence of bedbug infestations, and the warm temperatures that characterize the area. Consistent with findings from our earlier ‘open space’ research with the same communities, ownership of LLIN is not synonymous with their use [11]. In the Ruhuha sector, agriculture is the common source of income since the area is rural and surrounded by a lake. The two geographic characteristics may be linked with the use of LLIN in daily agricultural and fishing activities for some community members, especially in circumstances where malaria is not perceived as serious disease and/or nets are freely provided.

Provision of additional LLIN as suggested by participants in this study may be of considerable importance to those who do not possess them but may not be justified for households that use LLIN for purposes other than malaria prevention. While it is important for the government of Rwanda to minimize access barriers for vulnerable populations such as pregnant women and children and ensure the universal coverage of LLIN, tackling the question of how to enhance correct use of LLIN is regarded as a priority. As reported elsewhere, the promotion of LLIN usage has become a key malaria control strategy since it has been proven to be cost effective [13,14]. In our context, promoting LLIN usage must be accompanied by measures to control bedbugs. The World Health Organization (WHO) suggests that bedbugs contribute to the indirect ineffectiveness of malaria control measures and recommends the use of insecticides such as pyrethroids, diazinon, bendiocarb and dichlorvos [15]. And indeed, similar insecticides (pyrethroids and bendiocarb) are being utilized during IRS at least twice a year in the Ruhuha sector. However, special attention should be given to mattresses, furniture, as well as cracks and crevices in walls and floors to provide optimum results and increase acceptability of IRS. This approach may require a review of the goals and budget for IRS to incorporate considerations for factors that appear more important to local households, such as elimination of not just mosquitoes, but also other household pests that appear to affect the acceptability of and adherence to malaria control interventions.

Utilization of other community-based initiatives may also be effective. Rwanda has an innovative monthly community works program locally known as “*umuganda*” that supports cleanliness as well as act as a platform in which the local community gather and share relevant information about development, health and security. In a bottom-up approach, “*umuganda*” can also be used as a channel to disseminate malaria-related messages and initiate collaborative activities. For example, communities may organize a campaign for fighting bedbugs in villages most affected and/or plan for plastering the houses of families with poor housing conditions. The focus of “*Umuganda”* in regards to destroying mosquito-breeding sites around homesteads has been promoted and should be maintained as the regulation of environmental conditions through management practices at community level could reduce disease burden [16].

Among the preventive measures applied at the community level, IRS was widely known by the participants. From an empirical perspective, IRS has proven to be effective in reducing malaria transmission [17,18]. However, similar to other settings in Uganda and Mozambique, some concerns were raised about cultural norms related to privacy and doubts about the efficacy of the control method [19,20]. IRS with pyrethroids (Deltamethrin) was performed two months before this study took place in Ruhuha [5]. However, the method was generally perceived as ineffective and thought to cause proliferation or activation of other insects in the households rather than killing them. On one hand, the findings suggest possible resistance of mosquitoes against this insecticide as highlighted elsewhere [15]. Further entomological research is needed to establish the efficacy of IRS in Rwanda considering that community sensitization can only be effective if factors underlying community concerns are clearly understood and verified. On the other hand, the partial coverage (some rooms exempted) coupled with a lack of adherence to the instructions given for IRS reported by some households suggest that a behavioural change response should be a goal. This approach would require more efficient sessions of IRS education at household level where all rooms of the house need to be sprayed for efficacy.

At the individual level and considering the local population’s knowledge of malaria symptoms, other studies have yielded similar findings to this one, whereby malaria symptoms were related to illness in the community [21-23]. For example, severe malaria was reported to be associated with convulsions that were misinterpreted as witchcraft, which involved seeking treatment from ethno-medicine. This finding suggests that additional information and messages about the symptoms of severe malaria should be given to the community to prevent that some patients seek traditional medicine. Second, collaboration with ethno-medicine practitioners to initiate early referral for presumed malaria cases should be considered, although this can be seen as challenging since it could go against the direct interest of practitioners.

With respect to health care seeking behaviours, the main choice made by several members of the community was the official health centre since they could utilize their CBHI or CHWs for children below five years old. This finding of the independent and significant association between utilization of health services and membership in CBHI is in accordance with other research findings in Africa [24]. However, findings from the participants’ self-reports underscore a drop in the number of people having a CBHI due to the socio economic categorization in Rwanda piloted since 2011. Conversely, the local leaders did not acknowledge the required amount of families’ contributions as a barrier for joining CBHI, but rather focused on the benefits of CBHI. A possible reason for this may be that local authorities are expected to promote the uptake of government policies by the community. Nevertheless, a review of the socio-economic categorization is needed and this has been acknowledged at the national level. This review should consider the implementation process and the characteristics of categorization to classify the community into proper categories and, therefore, the people’s ability to contribute to the CBHI scheme instead of resorting to self-treatment. Self-treatment is defined in this context as using over-the-counter medicines, or herbal medicines or visiting a traditional healer. Though these choices were attributed to a few members of the community as also suggested elsewhere [25], the involvement of relevant stakeholders working in these sectors is a crucial step in malaria elimination. The findings of this study, mainly from FGD with health professionals, corroborate what has been previously reported by Ingabire et al. in 2014 [11] for the rationale of training owners of private drugstores and traditional healers in symptoms recognition and suggesting that they provide referrals to the health centre for malaria optimal healthcare.

Malaria elimination in Europe and North America required improvement of socio-economic conditions and the longer-term education for a significant decline in malaria [26]. Therefore, there is a need to sensitize the local communities to fight against poverty since this is strongly related to malaria by grouping the community into cooperatives that generate income, thus improving the members’ livelihoods. Furthermore, targeting students in schools for malaria messages can mean that the message will be applied in their homes and spread among other family members, which could have a great impact in Ruhuha settings. This is similar to what has been found in Thailand [27], where a study demonstrated how school children acted as messengers for various community-based anti-malaria actions. The actions included developing and issuing newsletters and leading information, education and communication sessions.

The current study provides new insight in regard to malaria and recalls some of the subjects highlighted in the previous study by Ingabire *et al.* [11]. Both studies illustrated the community’s and various stakeholders’ perceived willingness to engage in malaria elimination and reported misuse of preventive measures. However, the current study enabled researchers to interact with a variety of community members, purposively selected for their role in Ruhuha community. Detailed social and health complexities revealed by this study in relation to malaria preventive measures underpinned the great priority for continuous efforts to further reduce malaria transmission in the area as well as in similar settings.

In view of the above-mentioned findings from both studies, MEPR initiated interventions including, notably, the engagement of multi-sectoral stakeholders through the establishment of community malaria actions teams (CMATs), at the village level. Thirty-five teams, each consisting of a village leader, a community health worker and a youth member, represent 35 villages (one team per village) in the Ruhuha sector. The teams are expected to work closely with the community to identify malaria-related problems and find solutions, hence addressing the factors that have been identified as impeding the acceptability and use of malaria preventive measures. The approach is promising and further studies are planned to evaluate the outcome.

## Conclusion

Critical factors for this malaria elimination project to address based on the findings of this qualitative study include reduction in LLIN use due to bedbug infestation and hot climatic conditions, negative community perceptions of the effectiveness of IRS, and delays in health care seeking. Targeted interventions at the individual, community, and policy levels are needed to achieve optimal gains from available preventive tools. Additional tools based on the local ecology such as the community involvement in larval source control could also supplement the existing strategies and accelerate the progress in malaria reduction in this area of Rwanda.

### Consent

Informed consent was obtained from the participants for the publication of this report and any accompanying images.

## Abbreviations

CHWs: Community health workers
CBHI: Community based health insurance
FGD: Focus group discussion
LLIN: Long lasting insecticidal nets
IRS: Indoor residual spraying
ACT: Artemisinin-based combination therapy
NMCP: National malaria control programme
WHO: World Health Organization
